# Electro-mechanical dynamics of spiral waves in a discrete 2D model of human atrial tissue

**DOI:** 10.1371/journal.pone.0176607

**Published:** 2017-05-16

**Authors:** Paul Brocklehurst, Haibo Ni, Henggui Zhang, Jianqiao Ye

**Affiliations:** 1 Engineering Department, Lancaster University, Lancaster, United Kingdom; 2 Biological Physics Group, School of Physics and Astronomy, University of Manchester, Manchester, United Kingdom; Universiteit Gent, BELGIUM

## Abstract

We investigate the effect of mechano-electrical feedback and atrial fibrillation induced electrical remodelling (AFER) of cellular ion channel properties on the dynamics of spiral waves in a discrete 2D model of human atrial tissue. The tissue electro-mechanics are modelled using the discrete element method (DEM). Millions of bonded DEM particles form a network of coupled atrial cells representing 2D cardiac tissue, allowing simulations of the dynamic behaviour of electrical excitation waves and mechanical contraction in the tissue. In the tissue model, each cell is modelled by nine particles, accounting for the features of individual cellular geometry; and discrete inter-cellular spatial arrangement of cells is also considered. The electro-mechanical model of a human atrial single-cell was constructed by strongly coupling the electrophysiological model of Colman *et al*. to the mechanical myofilament model of Rice *et al*., with parameters modified based on experimental data. A stretch-activated channel was incorporated into the model to simulate the mechano-electrical feedback. In order to investigate the effect of mechano-electrical feedback on the dynamics of spiral waves, simulations of spiral waves were conducted in both the electromechanical model and the electrical-only model in normal and AFER conditions, to allow direct comparison of the results between the models. Dynamics of spiral waves were characterized by tracing their tip trajectories, stability, excitation frequencies and meandering range of tip trajectories. It was shown that the developed DEM method provides a stable and efficient model of human atrial tissue with considerations of the intrinsically discrete and anisotropic properties of the atrial tissue, which are challenges to handle in traditional continuum mechanics models. This study provides mechanistic insights into the complex behaviours of spiral waves and the genesis of atrial fibrillation by showing an important role of the mechano-electrical feedback in facilitating and promoting atrial fibrillation.

## 1 Introduction

Abnormalities in cardiac electromechanical activities associated with cardiac arrhythmias may have catastrophic consequences, leading to sudden death [1]. The most prominent cardiac arrhythmias are related to the ventricles, but atrial fibrillation (AF), while not as immediately deadly, is also a major clinical problem [2, 3]. AF has been associated with significant public health consequences, including reduced quality of life of the patients, increased hospitalization rates and medical costs [2]. However, current treatment of AF is unsatisfactory due to incomplete understanding of the mechanisms underlying the initiation and maintenance of AF, and how the abnormal electrical excitation impairs cardiac mechanical contraction.

Computer models of cardiac electromechanical functions can be used as a powerful tool to investigate the underlying mechanisms of AF [4] in a detailed way that is difficult to implement in experimental/clinical settings, given the complex nature of the heart [5]. Biophysically accurate modelling of the electro-mechanical dynamics of the heart is still a challenge, however, owing to the intrinsic heterogeneity in electrophysiology, anisotropy in anatomical structure and nonlinearity in its functions [6].

The mechanical contraction of myocardium influences the electrical behaviour of the heart, via a mechano-electrical feedback (MEF) [7]. This feedback process occurs through multiple mechanisms, including velocity-dependent cross-bridge kinetics of myofilament [8], length and tension-dependent binding rates of calcium to Troponin C [9], and a stretch-activated channel in the sarcolemma membrane [10]. MEF may have both pro- and anti-arrhythmic consequences, but mechanisms underlying these consequence have not been well elucidated yet [11]. Examples include “commotio cordis” and “precordial thump”, demonstrating that chest impact can both cause and stop arrhythmias [12, 13]. The action potential of myocytes may be significantly altered in response to stretch [14]; such effects can be significant and are an important direction of research in cardiac electrophysiology [15]. For a biophysically detailed computer model of the human atria, it is necessary to include MEF. Recently, some studies have been conducted to investigate the effect of MEF on cardiac electrical and mechanical behaviours [11, 15], including our previous studies [4, 16]. It has been shown in a discrete bi-domain model of a 1D fibre that MEF slowed down excitation wave conduction and caused conduction block [17]. In an electro-mechanical model of the left human ventricle, MEF was found to induce instability to stable spiral waves [11]. In a discrete mass-lattice model of ventricular tissue [15], it was found that the stretch-activated current affected the core size, period and tip meandering of spiral waves. However, the influence of MEF on the dynamics of spiral waves in the atria is still incompletely understood [11, 18].

It has been shown that persistent atrial arrhythmias lead to alterations in the physiological properties and functions of cardiac tissue [2, 3], which is termed as AF electrical remodelling (AFER). AFER includes changes in the density and kinetics of membrane ion channels which may increase the propensity for temporal AF to evolve into chronic AF (AF “begets” AF) [3]. AFER also affects the intracellular calcium handling processes [2, 3, 19], which may impair cardiac mechanical contractions. However, the effect of the AFER on cardiac electro-mechanics has not been fully elucidated. A recent study [4] is one of the first to address this, in a 3D electro-mechanical model using continuum mechanics. It was shown that AFER impaired tissue’s contraction power, which might be responsible for weak atrial contraction as seen in atrial stunning after cardioversion [4, 20].

In the vast majority of cardiac electro-mechanical models, traditional uniform and continuum mechanics has been assumed [4, 6, 11, 18]. However, such an assumption has significant limitations for the atria as it neglects the essential intrinsic properties of the atrial tissue including the tissue’s electrophysiological heterogeneity, anisotropic structure and discrete cellular spatial arrangement. First, atrial tissue is anisotropic in structure, with cells being arranged into fibers, forming primary conduction pathways. Second, it presents features of discrete cell spatial arrangement and discontinuous microstructure, which may cause discontinuous wave front of excitation waves that may play important roles in in the genesis of cardiac arrhythmias [21]. Such intrinsic atrial properties are often not taken into consideration in continuum methods, which use a spatial resolution far higher than that of a single atrial cell. Instead, a discrete element method (DEM) approach [22] provides a suitable way to capture the intrinsically discrete nature of cardiac tissue.

Cardiac arrhythmias, including AF, are associated with re-entrant excitation waves that are analogous to spiral wave solutions in an excitable system [23]. The presence of spiral or scroll waves during fibrillation has been confirmed experimentally [24, 25]. In this paper, we apply the discrete electro-mechanical model developed in [22] to investigate the effects of the MEF on the dynamics of spiral waves in the human atria. To construct our model, we first modified the single cell model of the human atrial cell developed by Colman *et al*. [26] to incorporate the modified Rice *et al*. [27] myofilament model describing the activation and force generation in cardiac myofilament. The electro-mechanical coupled model was validated by reproducing the experimental time courses of the electrical and mechanical activities of atrial myocytes. The single cell model was then incorporated into a coupled network of cardiac myocytes to investigate the functional impacts of the MEF on the dynamics of spiral wave in normal and AFER tissue.

## 2 Method

The model used to represent human atrial tissue spans two scales: the single-cell and multicellular tissue scales, which are introduced as follows.

### 2.1 Single-cell model

#### 2.1.1 Modelling the cellular electrophysiology

For modelling atrial cellular electrophysiology, the electrophysiological model for human right atrial cells from Colman *et al*. [26] is used. To simulate the AF-induced electrical remodelling (AFER) on variant ion channels, we followed the approach of Colman *et al*. [26] to consider variant experimental data sets on AFER.

In the cell model, AFER reduces [*Ca*^2+^]_*i*_, leading to a decreased sarcomere length shortening and reduced active force in the mechanical model (see Section 2.1.2). As compared to the non-AFER reference state, different parameter sets of AFER (AF1, AF2, AF3 and AF4) in the Colman *et al*. [26] study produced a reduction in active force of 94%, 92%, 83% and 92%, respectively. Experimental data shows that in AF patients the average force of atrial contraction is reduced by around 75% [28], and hence in this study we used the AF3 parameter set as described in *Colman et al*. [26].

#### 2.1.2. Modelling the development of active force

To simulate the active force generated at the cellular level in response to electrical action potential and the intracellular [*Ca*^2+^]_*i*_, the Rice *et al*. [27] model of rat ventricular myofilament dynamics is modified to fit experimental data obtained from human atria [29].

**Fig 1:**
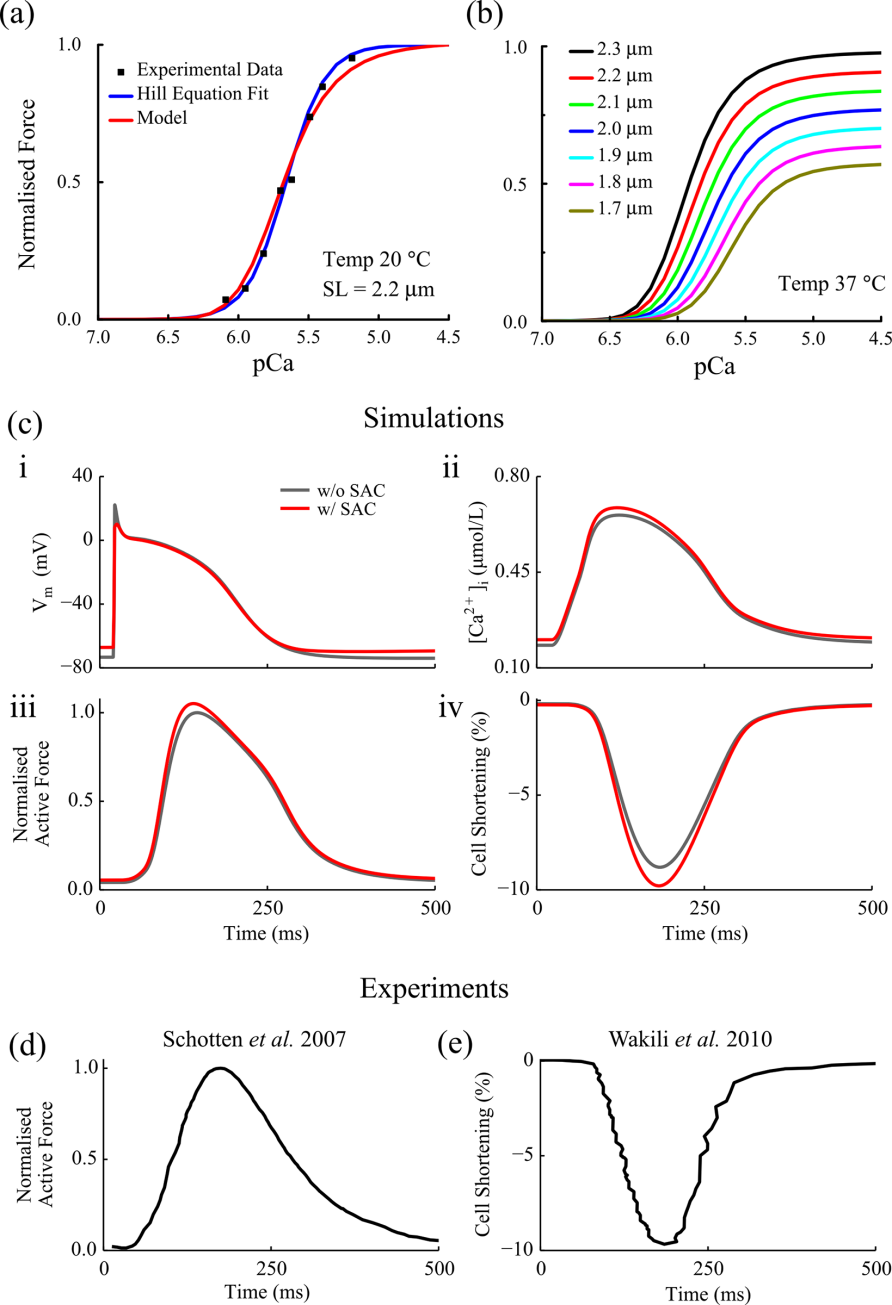
(a) Comparison between experimental and simulated data of the [*Ca*^2+^]_*i*_- tension relationship in human atrial cells, pCa = −*log*_10_[*Ca*^2+^]_*i*_; experimental data were digitalised from *[29]*. The active forces are normalised to the force at pCa = 4.5. (b) Simulated active forces at various fixed sarcomere lengths. The forces are normalised to the maximum force of the cell produced at SL = 2.3 μmin the model. (c) Simulated time courses of (i) AP, (ii) calcium transient, (iii) active force and (iv) SL shortening without *I*_*SAC*_ (grey), and with *I*_*SAC*_ (black). (d) Experimental data of time course for active force produced by human atrial myocytes *[34]*; the curve is normalised to the maximal value. (e) Experimental data of time courses for cell shortening observed in canine myocytes [35], showing around 10% cell shortening as seen in simulation (c(iv)). The experimental traces were digitalised from the corresponding original publications as labelled on top of panels (d-e).

The isometric protocol has been used to characterise the steady-state active force-Ca^2+^ relationship in human cardiac myocytes/myocardium at fixed cell/trabecule length [27, 29]. To account for the human atrial specific force-Ca^2+^ observed experimentally, a number of parameters governing the myofilament dynamics (the regulatory [*Ca*^2+^]_*i*_ binding to troponin, the cooperativity of the regulatory unit and the cross-bridge cycling) in the Rice *et al*. model [27] are optimised to reproduce the experimental steady-state force-Ca^2+^ relationship observed at room temperature using the isometric protocols [27, 29] (Fig 1(A)). In the parameter optimisation, the squared root difference between the simulated and experimental force-Ca^2+^ relations [29] using the isometric protocol is defined as the cost function, and is subsequently minimised using the Nelder-Mead Simplex algorithm [30] built in the *scipy-optimize* package, an open source optimisation library as part of the *SciPy* ecosystem [31]. The resulting parameters are listed in Table 1, in which they are compared to the original parameters of the Rice *et al*. model [27] representing the myofilament dynamics of rat ventricular cells. Detailed definitions of these parameters can be found in [27].

**Table 1:** Modifications to the Rice *et al*. myofilament model parameters to simulate the active force development in the human atrial cell. Parameters are defined in *[27]*.

Rate Constant	Original value [27] Species: Rat	Fitted value Species: Human
*k*_*on*_ (ms^−1^μm^−1^)	50	87.7
*k*_*offL*_ (ms^−1^)	250 × 10^−3^	397.2 × 10^−3^
*k*_*offH*_ (ms^−1^)	25 × 10^−3^	39.7 × 10^−3^
*perm*_50_	0.5	0.99
*n*_*perm*_	15	7.67
*f*_*app*_ (ms^−1^)	500 × 10^−3^	249.7 × 10^−3^
*g*_*app*_ (ms^−1^)	70 × 10^−3^	28 × 10^−3^
*h*_*f*_ (ms^−1^)	2000 × 10^−3^	998.9 × 10^−3^
*h*_*b*_ (ms^−1^)	400 × 10^−3^	160.1 × 10^−3^
*g*_*xb*_ (ms^−1^)	70 × 10^−3^	28 × 10^−3^

Fig 1(A) illustrates the simulated force-Ca^2+^ relation using the myofilament model with the fitted parameters, which is compared to the experimental force-Ca^2+^ relation [29]. Fig 1(B) shows the active force plotted against Ca^2+^ at various sarcomere lengths (SL): the force-Ca^2+^ relation is shifted to the left for higher SLs, indicating the pronounced dependence of active force on the SL. The temperature dependent rate constants are corrected to the physiological temperature using the temperature correction functions native to the Rice *et al*. model [27]. Close match between the model simulation and experimental data of the force-Ca^2+^ relation validated the use of parameters of the myofilament model for human atrial cells.

The mechano-electrical coupling is achieved by coupling the intracellular calcium transient [*Ca*^2+^]_*i*_ produced by single cell electrophysiological model into the updated Rice *et al*. myofilament model. To model the active force development of myocytes during an action potential, the isotonic stretch protocol is adopted: an external constant force is applied so that the SL of the cell is slowly stretched from a resting length of 1.9 μm to 2.2 μm, reaching an equilibrium state. The electrical stimuli are then applied to give rise to action potentials.

#### 2.1.3. Inclusion of stretch-activated channel current

The Colman *et al*. model is further modified to incorporate a stretch activated channel (SAC) current, which contributes to the MEF. SAC is a type of mechanically activated ionic channels, exhibits direct modulations on action potentials of myocytes in a strain-dependent manner. In accordance with previous studies [16, 32, 33], *I*_*SAC*_ is modelled using the following formulation:

$$I_{SAC}=G_{stretch}P_{m}\left(V_{m}-E_{stretch}\right),$$ (1)

where *G*_*stretch*_ and *E*_*stretch*_ are the maximum conductance and reversal potential of the channel, respectively, and *P*_*m*_ is the normalised open probability of *I*_*SAC*_, given by:

$$P_{m}=\frac{1}{1+\exp\left(-\left(\varepsilon -\varepsilon_{half}\right)/K_{\varepsilon }\right)}.$$ (2)

Here, ε is the engineering strain (linearly dependent on the stretch), *ε*_*half*_ is the half activation strain, and *K*_*ε*_ is the activation slope. In this study, *G*_*stretch*_ = 0.0061 ms/μF; *E*_*stretch*_ = −1 mV [16]; *ε*_*half*_ = 0.163 [32]; *K*_*ε*_ = 0.0266. *I*_*SAC*_ is assumed to be equally permeable to three major ions: sodium (Na^+^), potassium (K^+^), and calcium (Ca^2+^).

As shown in Fig 1(C), inclusion of *I*_SAC_ depolarises resting potential (from −75 mV to −68 mV), reduces the overshoot of the action potential, and slightly increases the diastolic and systolic levels of the intracellular calcium transient, leading to increased amplitude of active force and thus cell shortening. The impact of *I*_SAC_ on AP is concordant with previous studies in atria [36] and ventricles [16].

The electro-mechanical model successfully reproduces the characteristics of the time courses of active force development and cell shortening. The model is validated by simulating calcium-tension relationship at various sarcomere lengths under isometric condition, as shown in Fig 1A and 1B. The updated myofilament model successfully reproduces the dependence of the active force on the sarcomere length as seen in cardiomyocytes. The time courses of simulated active force and cell shortening during AP are qualitatively comparable to the results reported in a number of experimental studies [34, 35, 37, 38] in canine atrial cells as shown in Fig 1D and 1E. Though there is no experimental data from human atrial cells that are available to compare with, our model produces cell shortening of around 7–10% depending on parameters and region, which is quantitatively comparable to previous experimental studies on canine atria showing the cell shortening ranges from approximately 4% to 10% (4% in [34], 7.8% in [39] and 10% in [35]), which justifies the use of model parameters for myofilament dynamics.

### 2.2. Tissue model

#### 2.2.1.Mechanical model

To couple multiple cells together, we use the DEM method introduced in [22], which shows advantages over continuum mechanics models for simulating the discrete and inhomogeneous nature of the atria. In the DEM model, the basic elements are particles that may be bonded together using contacts to model elastic materials. DEM is a dynamic method, which tracks the velocity and position of each particle by solving explicit equations for their translational and rotational motion. It may also allow considerations of the interaction of individual cells arranged in a biophysically detailed structure, in both an electrical and mechanical context.

**Fig 2:**
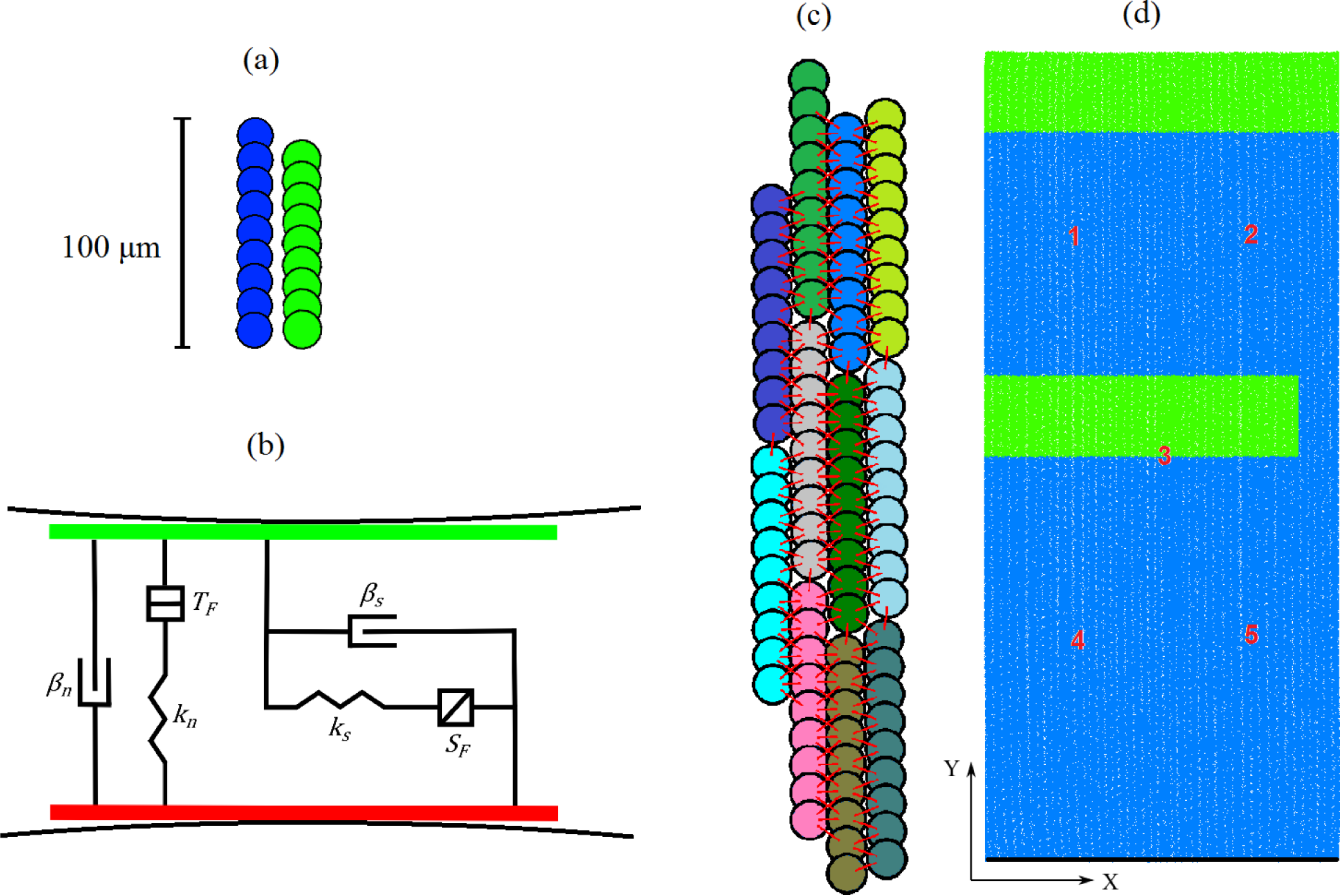
Representation of cells and tissue using DEM particles. (a) A single cell comprised of 9 particles in its initial (blue) and fully contracted (green) states. Note each state has identical two-dimensional area, to satisfy incompressibility of cardiac tissue. (b) The contact plane between two particles, with a spring and dashpot in the normal and shear directions. *k*_*n*_ and *k*_*s*_ are the normal and shear spring stiffnesses, *β*_*n*_ and *β*_*s*_ are the dashpot normal and shear critical damping ratios, and *T*_*F*_ and *S*_*F*_ are the tensile and shear strengths of the contact under force [22]. (c) Demonstration of how clumps are bonded to their neighbours, creating a tight spatial distribution of particles representing atrial fiber tissue. Here, clumps are individually coloured and red lines represent a DEM contact bond. (d) The DEM distribution on which spiral investigations are conducted. DEM particles are green and blue, with green representing which regions receive external stimulus. The red numbers labelled cells for which AP data is saved for further analysis. The black line indicates which cells have mechanical boundary conditions applied.

Following [22], each cell is represented by a clump of nine overlapping particles, with the dimensions equivalent to an isolated atrial cell (initial length 100 μm and diameter 16 μm [40]). We can dynamically change the particle positions to simulate the contraction of a cell. This is performed using the mechanical element of the single-cell equations, and allows the cell’s contraction process to be modelled in DEM [22]. As atrial tissue is incompressible, the clump/cell’s volume must be constant throughout the lengthwise contraction, and this is achieved by modifying the particle radii using a geometric equation [22]. Fig 2(A) shows a single atrial cell represented using DEM, in its initial and contracted states. Fig 2(B) shows a visual representation of the contact plane between two particles. Contact bond parameters are chosen in an attempt to capture the elastic behaviour of atrial tissue [22]. An illustration of contact bonds between neighbouring myocytes in tissue is given in Fig 2(C). The constructed 2D tissue is shown in Fig 2(D). In order to prevent excess motion of the tissue as a result of contraction, we apply a mechanical boundary condition to the bottom row of cells. These cells are fixed in the y (vertical) direction, but are allowed to move in the *x* (horizontal) direction to allow as natural a response as possible. In simulations, the velocity and position of each particle are tracked by solving explicit equations for their translational and rotational motion, which has been detailed in [22].

#### 2.2.2. Electrical model

To allow electrical waves to propagate through the DEM particle distribution, the cells are electrically coupled. Each cell is equipotential and has a membrane potential *V* (mV), which is given by:

$$\frac{dV}{dt}=-\frac{\left(I_{ion}+I_{ex}+I_{n}\right)}{C_{m}}$$ (3)

where *I*_*ion*_ is the total ionic current (pA) of a cell and *C*_*m*_ the membrane capacitance (pF). The external stimulus *I*_*ex*_ is defined as:

$$I_{ex}=\{\begin{array}{l} S_{s,}\;\mathrm{if}\;S_{t}<t<S_{t}+S_{d},\\ 0,\;\mathrm{otherwise}, \end{array}$$ (4)

Here, *S*_*s*_ > 0 is the stimulus strength, *S*_*t*_ the time the stimulus is applied, and *S*_*d*_ the stimulus duration. The term *I*_*n*_ is a contribution from the neighbouring cells, representing electrical flow from cell to cell due to intercellular electrotonic coupling. Two cells are considered neighbours if there exists a DEM contact between them. If a cell labelled *H* with membrane potential *V*_*H*_ has *N* neighbours, the external stimulus for cell *H* is given by:

$$I_{n}=D\;\sum_{i=1}^{N}\left(V_{i}-V_{H}\right),$$ (5)

where *V*_*i*_ denotes the membrane potential of the neighbouring cell numbered *i*, and *D* is an electrical conductance parameter, simulating intercellular electrotonic interaction through gap junctions [41]. Gap junctions are predominantly present at the cell ends in the fibre direction, causing faster propagation along the fibres than the transverse direction. However, the physical contacts in this model are the DEM contacts between cells, which naturally are more numerous in the transverse direction. Following [22] we balance this discrepancy by assuming that even if multiple DEM contacts exist between two cells, the electrical flow is divided equally amongst them–that is, the contact surface area (and hence number of DEM contacts) between two cells does not affect the strength of conduction. The anisotropy in the tissue is naturally accounted for, due to the larger spatial step in the fibre direction: the model exhibits an anisotropy ratio of approximately 6:1, i.e., the same as the ratio of cell length to width [22]. Note that this also simplifies the formulation as we only need one conduction parameter, whereas typically continuum methods require the use of a diffusion tensor to achieve anisotropy.

### 2.3 Model construction for study the dynamics of spiral waves

We are interested in studying the functional impacts of the MEF on the characteristics of spiral waves using the described electro-mechanical model of human atrial tissue under the normal and AFER conditions. To do so, the model is applied to investigate four cases, which are referred to as follows:

- **M**–Full electro-mechanical coupling model without AFER
- **E**–Electrical-only model without AFER
- **MR**–Full electro-mechanical coupling model with AFER
- **ER**–Electrical-only model with AFER

The full electro-mechanical models include the stretch-activated MEF current, and tissue motion is calculated using DEM. The electrical-only models are performed on a static grid and do not include MEF.

A sufficiently large portion of tissue is required to initialise and sustain a spiral wave. For this, a model for a slab of tissue with 1.92 cm in width and 4 cm in height was constructed. This is done by arranging cells into aligned fibres, with randomly varying vertical offset (Fig 2(C)). We apply contact bonds to particles that have a surface gap (see [22, 42]) of 0.25*r* or less, with *r* being the particle radius. This value creates a dense network of mechanical contacts between particles (Fig 2(C)), facilitating a prompt tissue response to alterations in cell geometry and maintaining the incompressibility condition of atrial tissue [22]. The introduction of millions of contact bonds between particles initially causes contact force between particles, and subsequently particle motion. The system is computationally cycled until particle motion ceases and the DEM distribution reaches a stable equilibrium. There are 1200 cells in the horizontal direction and 400 in the vertical direction, resulting in a total of 4.32 million particles in the DEM distribution as shown in Fig 2(D).

To generate spiral waves, we use the typical S1-S2 protocol (Fig 2(D)). Cells in the upper green region receive a stimulus S1 at *t* = 1 ms. Cells in the lower green region receive a stimulus S2 at a later time which enables a spiral wave to initiate. The time *S*_*t*_ of S2 must be ascertained experimentally for each case, depending on the repolarisation refractoriness of the cells.

The same DEM distribution of tissue model is used for each model, in order to directly compare the results. The conduction parameter *D* = 600 nS is chosen and used for each model. The conduction velocity (CV) varies for each model and is reported in Section 3. We find that for higher values of *D*, the size of the tissue is not large enough to support sustained spiral waves for all models considered, owing to the large wavelength produced. Other parameter values for the model, including DEM parameters, are chosen as in [22].

### 2.4 Numerical methods

The ODEs of the single-cell model are solved using forward Euler integration combined with the Rush-Larsen scheme [43]. The calculation performed in the DEM alternate between the application of Newton’s second law to the particles and a force-displacement law at the contact. The computation cycle of the tissue model is a time stepping algorithm that consists of the repeated applications of the law of motion to each particle. The time-dependent equations are solved explicitly using a centred finite-difference scheme. The critical time step is estimated at the beginning of the cycle, as described in [42] using both a stiffness constraint and a kinematic constraint, and used to integrate the equations of motions through a 2^nd^ order accuracy velocity Verlet scheme for translational degrees of freedom, and a 4^th^ order accuracy Runge-Kutta scheme for the clump’s rotational degrees of freedom. The stability of the solutions is ensured if the time step does not exceed the critical time step calculated automatically by the software. The time step we have used in any cycle of the computation is Δ*t* = 0.004 ms, which is smaller than the default critical time step recommended by the software to ensure stability and convergence of the solution. This is verified by the fact that when we compare solutions of membrane potential and active force obtained from a local site of 2D tissue model (the middle of the tissue model) in control condition with that of a single cell model, a close match between them was observed. The full computational cycle for one time-step in the model is:

- For each cell, loop over neighbouring cells and calculate their contribution to that cell’s electrophysiology, *I*_*n*_;
- For each cell, solve the single-cell equations defining the electrical & mechanical behaviour of that cell;
- For each cell, update the particle radii and particle positions, such that the cell length matches the output of the single-cell equations;
- For each contact between particles, solve the force-displacement law, updating the contact forces based on relative particle motion and constitutive contact model;
- For each particle, solve the law of motion, updating the particle position and velocity due to contact forces.

The explicit nature of the algorithms means each stage of the computational cycle above may be accelerated using parallelisation. Computations are performed on a single desktop computer using an Intel Xeon 3.6 GHz CPU, and multi-threaded using all 8 threads. DEM calculations are performed using Itasca PFC version 5.0 [42], and all other calculations are performed using a custom C++ library interfacing with PFC. Further computational savings are made by disabling the contact detection phase of DEM during cycling, as no new contacts are generated throughout the simulation. The computations take approximately 336 hours (2 weeks) to generate a 4-second simulated episode.

The initial conditions of the ODEs for the single-cell models may have critical effects on the results of tissue models. To facilitate faster convergence, single cell models under the various conditions were paced for 100 beats at 1Hz to generate initial conditions, which were subsequently incorporated in tissue modelling. External stimuli have a strength of *S*_*s*_ = 2 nA and a duration *S*_*d*_ = 2 ms.

## 3 Results

**Fig 3:**
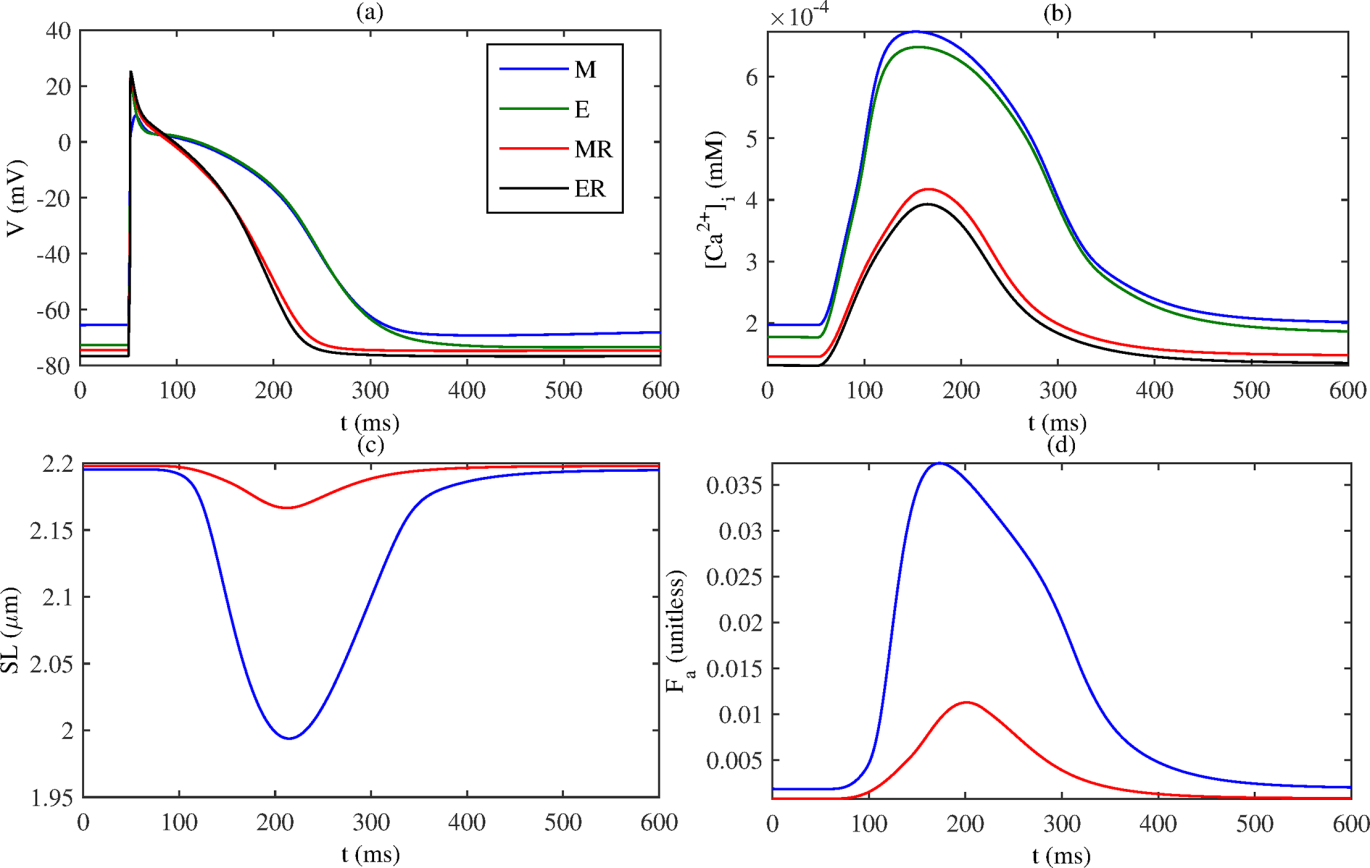
Simulation results in single cell model for each model: M (MEF, no AFER; blue), E (no MEF, no AFER; green), MR (MEF, AFER; red), ER (no MEF, AFER; black). (a) Membrane action potential *V*, (b) Intracellular calcium [*Ca*^2+^]_*i*_, (c) Sarcomere length *SL*, (d) active force *F*_*a*_.

First, we analyse the output of the single-cell model for the four cases considered: recall that mechanical models **M** and **MR** include the MEF and tissue contraction, whilst electrical models **E** and **ER** do not; and a suffix **R** indicates AFER conditions. Fig 3(A) shows the action potentials for each model. Comparing **M** (blue) and **E** (green), it is shown that the inclusion of MEF reduces the overshoot and upstroke velocity of the action potential. In addition, it depolarizes the cellular resting potential. Aside from these, the time courses of action potentials are similar between the two models (e.g. with a near-identical APD_90_). For the AFER models **MR** (red) and **ER** (black), inclusion of *I*_*SAC*_ has a similar effect, though not as profound an impact as seen in the normal tissue. In AFER conditions, the APD_90_ is considerably shorter than in the normal conditions, and the resting potential is more negative.

Fig 3(B) shows the [*Ca*^2+^]_*i*_ concentrations for each model after stimulus. AFER leads to a decrease in peak [*Ca*^2+^]_*i*_. Inclusion of MEF only results in a slight increase in [*Ca*^2+^]_*i*_. Fig 3(C) shows the time courses of sarcomere length shortening for the two mechanical models **M** and **MR**. AFER corresponds to a reduction in cell length contraction: for **M**, cell length contracts by 9.18% from resting, while for **MR** this is reduced to 1.43%. Fig 3(D) shows that the active force in the cell is markedly reduced for **MR**.

**Fig 4:**
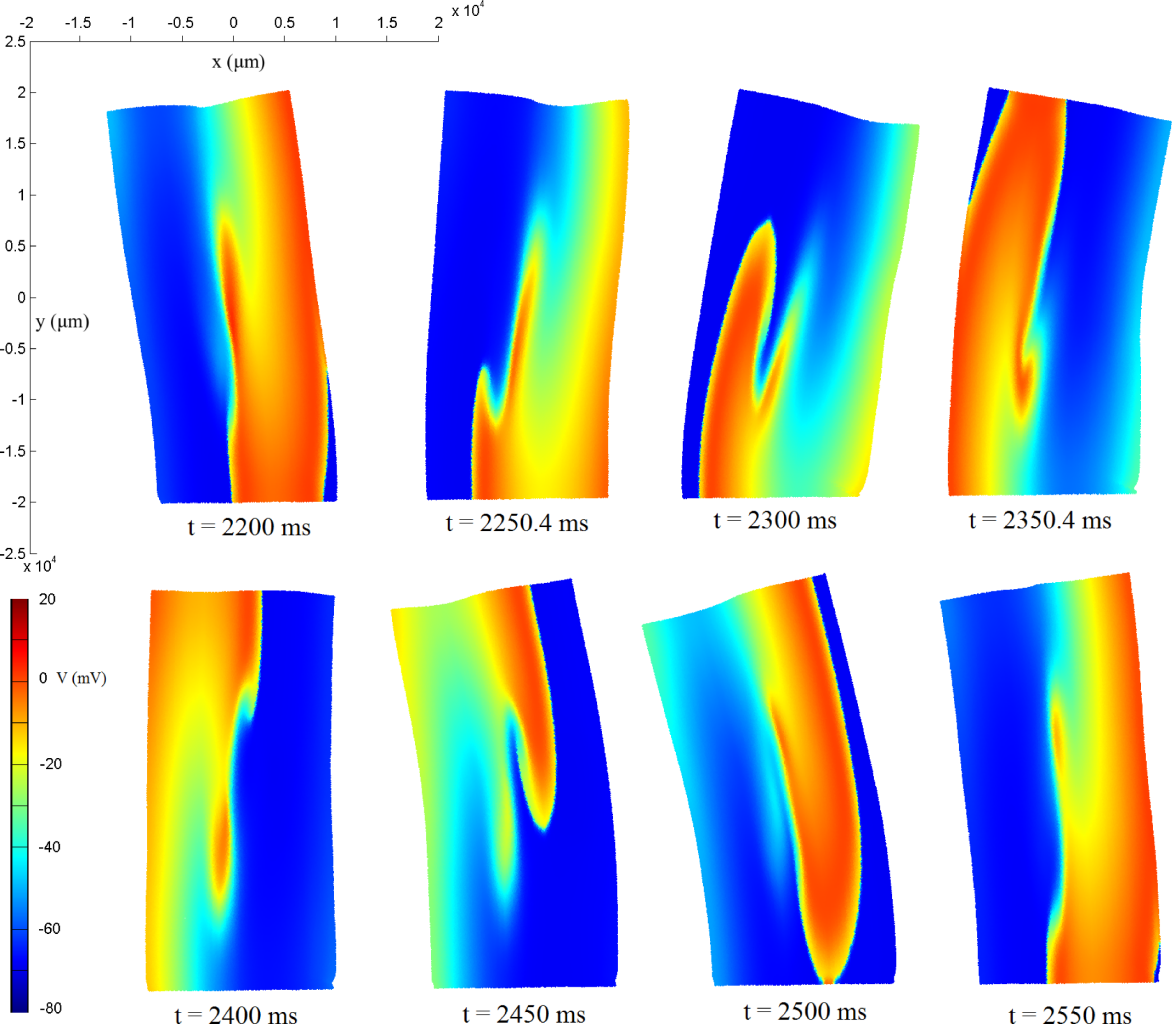
Snapshots of tissue deformation and membrane action potential conduction for model M during a spiral wave excitation in a 2D electro-mechanical coupling model of human atrial tissue (x, y: μm).

Spiral waves are generated for each model as described in Section 2.3. Fig 4 shows snapshots of the membrane action potential conduction throughout the tissue, for model **M** (full electro-mechanical model), as well as the evolution of tissue deformation. The spiral wave is successfully generated and maintained. The spiral rotates around the central core of the tissue. However, this central region is somewhat irregular in excitation. Frequently, a secondary wave-front is formed from the breaking up of the main spiral wave. This wavelet often self-terminates, further contributing to the spiral instability. Occasionally, the secondary wavelet reattaches with the primary spiral as it wraps around upon itself. This pattern is sustained throughout the lifetime of the spiral wave, becoming increasingly more pronounced with time, instead of stabilising. In general, the spiral wave meanders and rotates in an oval pattern, owing to the significant anisotropy due to the fibre direction. The spiral dynamics for each model are analysed in greater detail below.

Conduction velocity (CV) in the fibre direction is measured by timing how long the initial plane wave takes to reach the bottom of the tissue after the top region receives a stimulus (see Fig 2D). As the anisotropy ratio of the model is approximately 6: 1 (see Section 2.2.2), CV in the transverse directions are thus expected to be 83% slower than the quoted values: all CV values stated are for the fibre direction. The measured CV for each model is: **M**, 30.66 cm/s; **E**, 46.15 cm/s; **MR**, 46.51 cm/s; **ER**, 46.75 cm/s. Inclusion of MEF and AFER interact in different combinations to influence the CV in interesting ways. By comparing models **M** and **E,** we see that inclusion of MEF corresponds to a slowing down in conduction by 34%. Comparing models **M** and **MR**, for which MEF is present, the addition of AFER corresponds to an increase in CV of 52%. However, comparing **MR** and **ER,** for which AFER is included, addition of MEF has little effect on CV. Likewise, comparing **E** and **ER** where MEF is not included, addition of AFER has little effect on CV. In summary, model **M** (MEF, no AFER) has significantly lower CV than all other cases.

Usually AFER is characterised by a reduction in CV, whereas in our results AFER causes an increase in CV (comparing **M** and **MR**) or no change (comparing **E** and **ER**). Note that in the simulations, we did not consider AF-induced gap-junctional remodelling with the aim to highlight the functional impacts of AFER on electromechanics of atria. Such gap-junctional remodelling is believed to play a major role in the reduced CV at the tissue level. In our simulations, it was observed that ion channel remodelling caused an increase in maximal up-stroke velocity of the action potential (AP), as well as the overshoot. Such simulated effects of ion channel remodelling at the cellular level matched the clinical electrophysiological data of Dobrev *et al*. [44]. The increased maximal upstroke velocity and overshoot of APs consequentially led to an increased CV in the tissue level, in absence of consideration of gap-junctional coupling remodelling.

However, even without gap-junctional remodelling, some studies such as Zhang *et al*. [23] have shown that inclusion of AFER causes a decrease in CV, in contrast to our results. AFER has diverse effects on ion channels, with several experimental data sets available, each modifying different ion channels and by different amounts. To test the validity of the simulation, we have also considered the experimental data set from Bosch *et al*. [45]. By incorporating the Bosch *et al*. data into the model, we observed that the effects of AFER resulted in a more hyperpolarised resting potential, an increased up-stroke velocity and an increased overshoot, which were consistent with the clinical electrophysiological observation of Bosch *et al*. data. At the tissue level, such AFER produced a decreased CV, consistent with the study of Zhang *et al*. [23].

**Fig 5:**
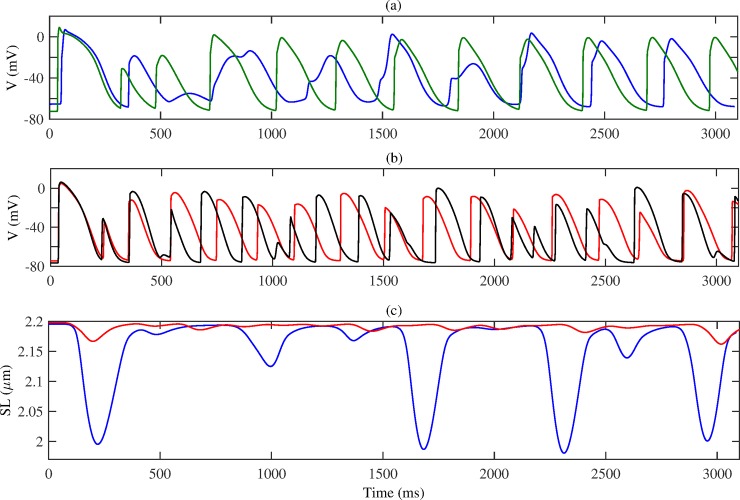
(a) The evolution of membrane action potential for the central cell in the tissue, throughout the time course of spiral wave excitation in the tissue. Here, model **M** is blue and **E** is green. (b) Membrane action potential for the central cell, for models **MR** (red) and **ER** (black). (c) The sarcomere length for the mechanical models **M** (blue) and **MR** (red).

In order to analyse the effects of MEF & AFER on the electrical and mechanical activities of cells in the tissue during spiral wave excitation, action potentials and sarcomere length are recorded in specific cells in the tissue (see Fig 2(D)). Fig 5(A) shows the time courses of membrane action potentials recorded from the central cell in the tissue (cell 3, as marked in Fig 2(D)) for models **M** and **E**. As the meandering of the spiral wave core interferes with the regular cycle of the excitation waves, deformed morphology of action potentials for some peaks are observed. When compared to Fig 5(B) showing the same for models **MR** and **ER**, more frequent excitation is observed, with smaller amplitudes of action potentials. Fig 5(C) shows the time course of the sarcomere length shortening during spiral wave excitation, for models **M** and **MR**. In both models the contractions are not consistent throughout time, with some being smaller than usual. As before, the AFER model **MR** continues to exhibit reduced contraction.

**Fig 6:**
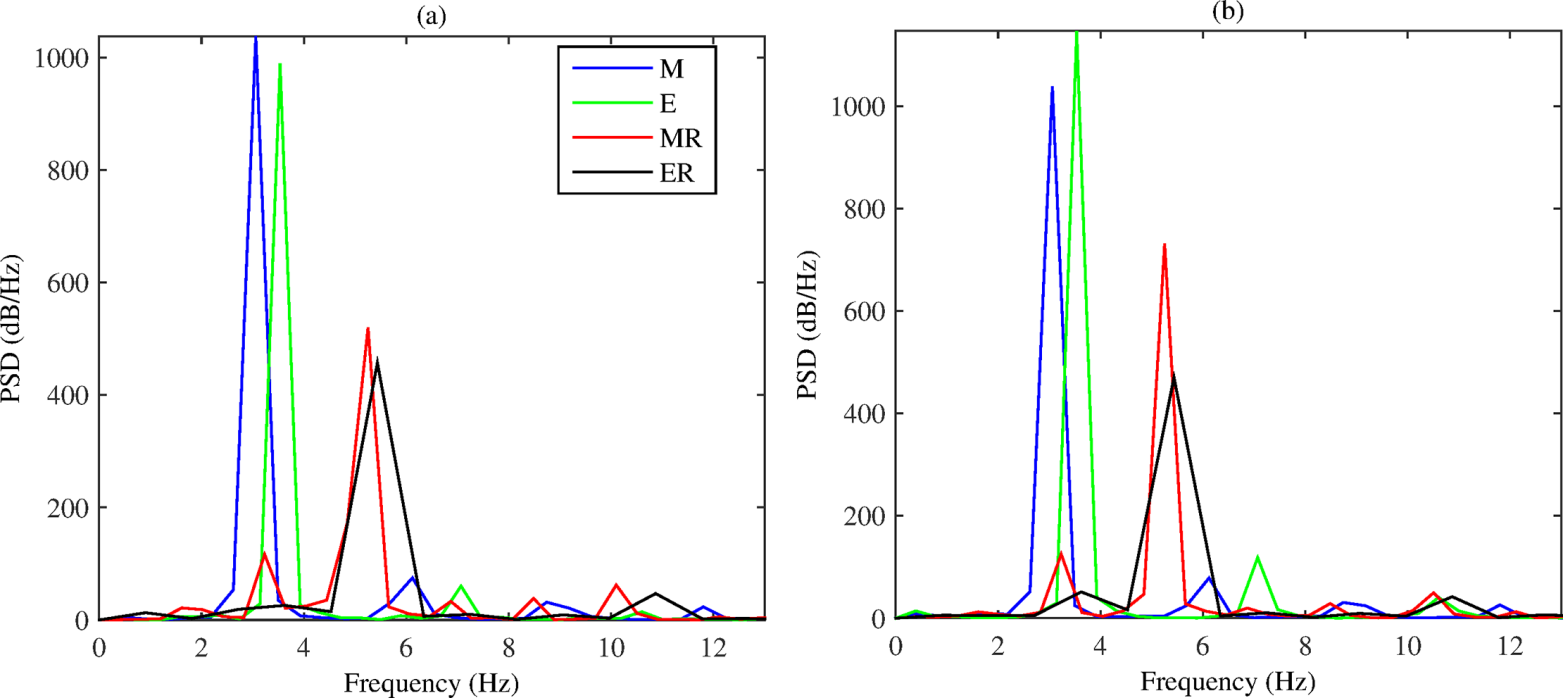
Frequency spectrum analysis for action potentials recorded from cell 1 (a) and cell 5 (b) in the tissue. In both graphs **M** is blue, **E** is green, **MR** is red and **ER** is black.

Fig 6 shows frequency spectrum analysis for action potentials recorded from cells 1 and 5 (as marked in Fig 2(D)) in the tissue. For each model, dominant frequencies are identified. From the frequency spectrum analysis, we calculate the power spectral density (PSD) of the recorded action potentials (recorded during the time period from *t* = 1000 ms to 3000 ms). For cell 1, in Fig 6(A), the full electro-mechanical model **M** exhibits the lowest dominant frequency of 3.06 Hz, followed by the electrical-only model **E** at 3.54 Hz. AFER models have higher dominant frequency: **MR** has dominant frequency at 5.25 Hz, whilst **ER** exhibits the highest at 5.44 Hz. AFER models **MR** and **ER** also have lower PSD. For each model, several other peaks occur, but with much lower PSD. Fig 6(B) shows the frequency spectrum analysis for cell 5, which shows similar results to those of cell 1, but with altered peaks of PSD. These results suggest that MEF accelerates the excitation rate of atrial spiral waves.

Further analysis is conducted to investigate the effect of MEF on the tip meandering pattern of spiral wave excitation. In continuum models of cardiac tissue, the location of the spiral “tip” is often determined by locating the intersection of the line *dV*/*dt* = 0 with a line of constant voltage *V* = *I* to give an exact point. In the present model, the membrane potential is not a continuous distribution and only exists for each discrete cell. Therefore, here we assign each cell a classification (i)-(iv) for the unique combinations of *dV*/*dt* > 0 or < 0, and *V* > *I* or < *I*. A discrete cell is a spiral tip if it is within a threshold distance of all four classifications. For models **M** and **E**, a value of *I* = −5 mV was used, and for models **MR** and **ER** a value *I* = −30 mV was necessary to locate all tips. Owing to the slightly unstable nature of the wave patterns towards the centre of the tissue discussed previously, for the non-AFER models **M** and **E** there are often multiple tips at any given time. In particular, when a secondary branch appears, it is calculated as an additional tip, though it often disperses or reattaches to the main spiral. Other times, the main tip disperses and is usurped by the secondary tip. The spiral tips are calculated for each time step and sorted into spiral trajectories throughout time. For clarity in the plots, only key spiral tips which last longer than 10 ms are plotted.

**Fig 7:**
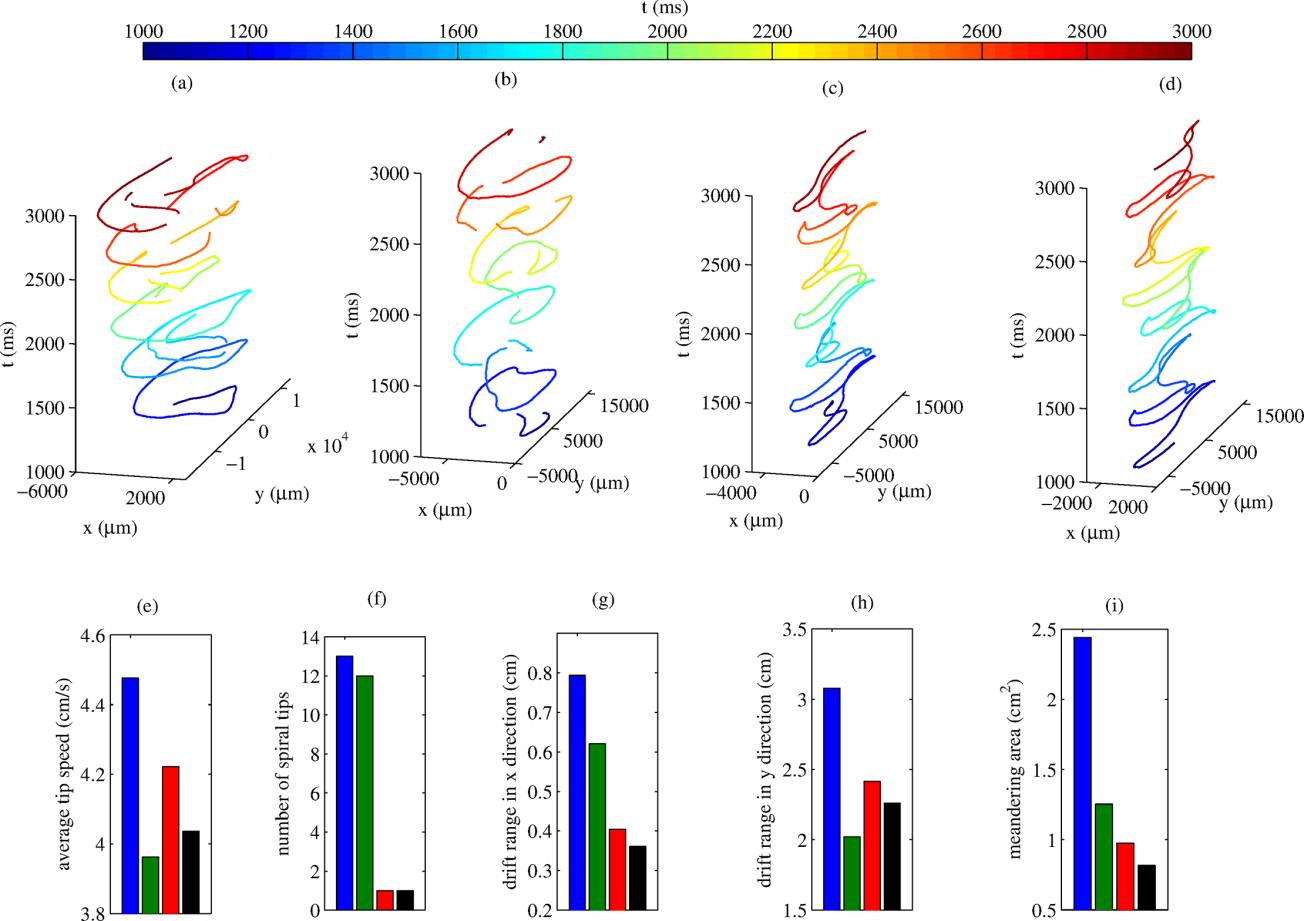
(a)-(d) Traced spiral tip trajectory for ***M***, ***E***, ***MR*** and ***ER***, respectively. Spatial location of the tip is given in the *x*−*y* plane, and the vertical axis (time) is coloured for clarity. (e)-(i) various properties of the spiral waves are displayed. In each graph **M** is blue, **E** is green, **MR** is red and **ER** is black.

The spiral tip trajectories for each model are shown in Fig 7A–7D. Further analysis of the tip trajectory behaviours is shown in Fig 7E–7I. For each model, the spiral tips exhibit substantial drift in both *x*–and *y*– directions, though drift in the fibre direction *y* was greater. Models **M** and **E** have disjointed spiral tip trajectories owing to the frequent wave-breaks (13 and 12 individual spiral tips respectively). However, for the AFER models **MR** and **ER**, there is a single spiral tip throughout the simulation. **M** has the highest average tip speed of 4.48 cm/s, with **E** having the lowest. **MR** has higher tip speed than **ER**, suggesting that the MEF results in a direct increase in spiral tip meandering speed. **M a**lso exhibits the highest spiral drift in both directions, resulting in the highest meandering area. Overall, **ER** exhibits the lowest meandering area.

**Fig 8:**
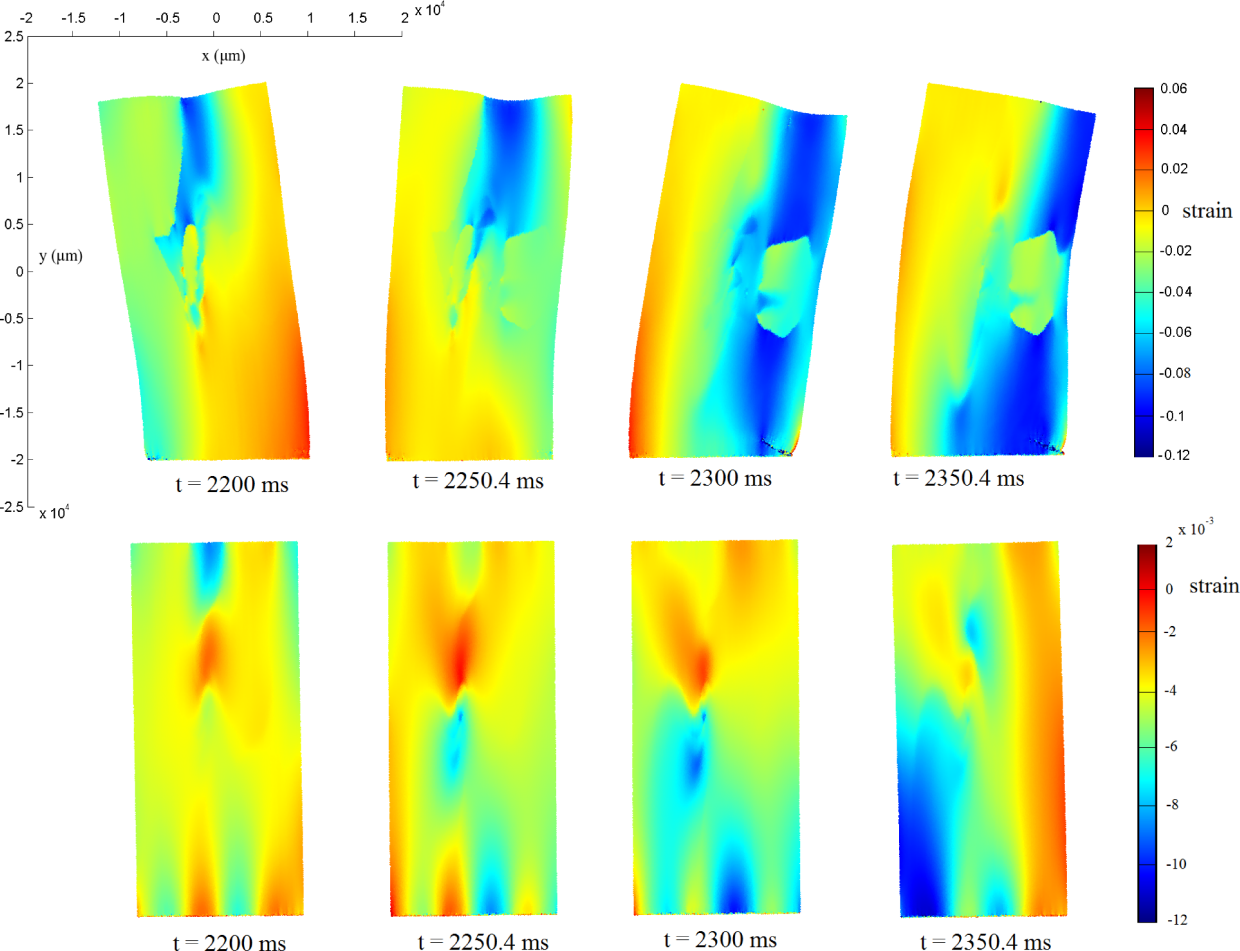
Estimated strain (unitless) in the fibre direction during spiral wave excitation. The top row shows the strain for **M**, and the bottom row shows the strain for **MR**. Note the different colour maps in each model.

We are interested in quantifying the distribution of strain throughout the tissue during contraction, to identify regions of high and low stretch. Formally, strain is a continuum measure and does not exist in a discrete assembly. However, we may calculate an estimate for the strain in the fibre direction at each discrete point, for the purpose of visualisation. This is achieved as follows: starting at the bottom cell in each fibre, calculate the reference distance between each cell and the next cell vertically along the fibre. The strain in the tissue at the point of this cell is estimated to be the current distance divided by the reference distance. The results of this analysis for **M** and **MR** are shown in Fig 8. For **M**, strain typically ranges from −0.12 to 0.06. The discrete nature of the assembly results in an occasional non-smooth distribution of strain, with ridges of sharp disparity developing, especially near the centre of the tissue. Regions of high absolute strain occur towards the edges of the tissue. **MR**, under AF-remodelled conditions, has notably less strain due to the reduction in cell contraction. Strain is typically between −0.0012 and 0.002. Regions of high absolute strain occur towards the upper-central portion of the tissue.

## 4 Discussion

### 4.1 Summary of major findings

A single cell model was presented for the electrical and mechanical behaviour of the human atria. The mechanical aspect was developed by updating the Rice *et al*. model [27] by modifying the parameters of the model to better fit human experimental data [29]. It was then coupled to the electrical model of Colman *et al*. [26] through calcium concentration and a stretch-activated channel current. The model is able to reproduce the action potential and intracellular *Ca*^2+^ concentration transient of human atrial myocytes [26], and the characteristics of the active force development and cell shortening that closely match to experimental data [34, 35], validating the model development.

The single cell model was then incorporated into a previously developed model of cardiac tissue using the discrete element method [22]. The tissue model was used to investigate the dynamics of spiral waves in 2D human atrial tissue. The nature of the model and spatial resolution allows us to account for the discrete spatial arrangement of individual cells and fibre direction. Its numerical efficacy permits a significant portion of tissue to be studied, which is sufficiently large to sustain spiral waves. By considering four different cases (**M**, a full electro-mechanical model; **E**, an electrical-only model; **MR**, full electro-mechanical with AFER conditions; **ER**, electrical-only model with AFER), we investigate the effect of MEF on the dynamical behaviour of spiral waves in normal and AFER conditions. Our results have shown that MEF slows down cardiac conduction, destabilises re-entrant spiral waves, decelerates excitation rate of spiral waves, increases the average spiral wave tip meandering speed, and increases the size of meandering area. It is also shown that AFER stabilises the spiral wave as well as drastically reduces the mechanical activity of tissue, owing to the lessened cell shortening resulting from reduced calcium transient. The discrete formulation of cells was observed to cause a discontinuous distribution of strain throughout the tissue, with ridges of disparity developing during peaks in tissue contraction.

### 4.2 Relevance to previous studies

This study is the first to investigate the effect of MEF and AFER on the dynamic behaviours of spiral waves in a discrete 2D model of human atrial tissue: some previous studies are particularly relevant but differ slightly. Kuijpers *et al*. [17] studied the effect of MEF on atrial excitation wave conduction in a discrete atrial model, though it was one dimensional; Keldermann *et al*. [11] studied MEF effects on spiral wave dynamics, though the model was continuum mechanics based and focuses on the ventricles; Colman *et al*. [26] introduced the cell model and AFER model we used here, but their work was focused on electrophysiological aspects; Weise *et al*. [15] used a discrete 2D model to investigate the effect of MEF on spiral waves, though it focused on the ventricles; and Adeniran *et al*. [4] studied AFER in a 3D electro-mechanical model, but uses continuum mechanics. Here we compare their conclusions with the present work.

In their discrete 1D fibre model, Kuijpers *et al*. [17] reported conduction slowing due to MEF under the normal conditions, which is in agreement with our results on CV. In addition we note that in the presence of AFER, MEF does not alter CV for a plane wave. Keldermann *et al*. [11] noted that MEF could induce the deterioration of an otherwise stable spiral wave into turbulent wave patterns. In agreement, we found that in general, the MEF spiral waves were less stable than those without MEF, both with and without AFER. The MEF spirals had higher tip meandering speed and area than their counterparts.

However, the present study found that the effect of AFER on spiral stability was more pronounced: AFER spirals were more stable compared to the non-AFER cases, both with and without MEF, sustaining a less turbulent spiral and more stationary spiral tip. Addition of AFER also caused lower meandering area and higher dominant frequencies both with and without MEF, which is in good agreement with Colman *et al*. [26] showing that AFER stabilised and accelerated spiral excitations in tissue. In another study, Adeniran *et al*. [4] found that at the cellular level AFER causes APD abbreviation and cell-shortening, and at the organ level, mechanical contraction was highly impaired, with which our results are in agreement.

Ostensibly the work of Weise *et al*. [15] is the most similar model to ours, though there exists many differences. For example, Weise *et al*. use a spatial discretisation of 2–5 times that of cell width, compared to the smaller particles used here. In addition, the model does not account for cellular geometry, nor the anisotropy in conduction or tissue spatial structural arrangement. Notably the work focuses on the ventricles/epicardium. The primary effect of stretch activated channel current was accommodation and pre-excitation of the medium. In addition, MEF caused an increase in drift, drift velocity and spiral wave period, which are in consistence with the present model.

### 4.3 Limitations and future work

Due to lack of experimental data from human atrial tissue, alternative experimental data from canine cells were used in validating the simulated time course of cell shortening elicited from APs. Since they are different species, the cell shortening of human atrial cells might differ from that of canine atrial myocytes, which may impose a limitation to the present study. However, the lack of specific experimental data of a specific species is a common challenge for cardiac modelling studies [26]. In line with previous modelling works [26, 40, 46–48] in which experimental data from alternative species were incorporated, the present study used canine data to validate the simulated cell shortenings. The electromechanical model may be improved if such data from human atrial cells are available in the future.

The advantages and limitations of the DEM model have been discussed in [22] and apply in the present study. Additionally, in the present study, the “clump” approach to modelling a single-cell saves computational time by skipping contact bonds within the clump: however, as a consequence each cell can only contract along its fibre direction, and cannot flex or bend orthogonally. Due to the microscopic size of myocytes, further investigation is needed on whether this has any effect when progressing to organ scale. Another limitation of the tissue simulations is that only myocytes are considered: the fibroblast may play a role in modulating the excitation wave propagation in AF [49] and thus warrants future investigations incorporating fibroblasts into the model. A third limitation is that the present paper focuses only on idealised tissue with aligned fibres, uniform cell types and contact bond strength, as the main focus was the comparison of spiral wave properties amongst the three cases. Future studies could incorporate more of the atria’s intrinsic structural and electrical heterogeneities.
